# Prevalence of anemia and its predictors among patients with chronic kidney disease admitted to a teaching hospital in Ethiopia: A hospital-based cross-sectional study

**DOI:** 10.1097/MD.0000000000031797

**Published:** 2023-02-10

**Authors:** Filagot Bishaw, Maekel Belay Woldemariam, Gashahun Mekonen, Bezawit Birhanu, Abinet Abebe

**Affiliations:** a Department of Internal Medicine, Faculty of Medicine, Institute of Health, Jimma University, Jimma Ethiopia; b Department of Health Policy and Management, Faculty of Public Health, Institute of Health, Jimma University, Jimma Ethiopia; c Department of Clinical Pharmacy and Pharmacy Practice, School of Pharmacy, College of Medicine and Health Sciences, Mizan-Tepi University, Mizan Ethiopia.

**Keywords:** anemia, chronic kidney disease, Ethiopia, predictors

## Abstract

Anemia is a common complication of chronic kidney disease (CKD) and is associated with adverse patient outcomes. However, data on the prevalence of anemia in CKD patients is sparse, particularly in resource-limited settings. Therefore, this study aimed to assess the prevalence of anemia and its predictors among patients with CKD admitted to the Jimma medical center, southwest Ethiopia. A hospital-based prospective cross-sectional study was conducted from September 1 to November 30, 2020. All adult patients with CKD aged ≥18 years who fulfilled the inclusion criteria were consecutively recruited into the study. Data were entered into the Epi data manager version 4.4.1 and then exported to SPSS version 22 (IBM Corp., Armonk, NY) for analysis. The predictors of anemia were determined using multivariable logistic regression analysis. Statistical significance was set at *P* < .05. A total of 150 patients were included in this study. Of these, 64.67% were male, 56.67% had stage 5 CKD, 78% had a CKD duration of less than 1 year, and 74% had proteinuria. Hypertension (40.7%) and diabetes (14.7%) were the common causes of CKD. The prevalence of anemia was 85.33%. Of the patients, 28.67%, 40.67%, and 16% had mild, moderate, and severe anemia, respectively. On multivariate logistic regression, stage 4 CKD (adjusted odds ratio [AOR] 3.2, confidence interval [CI]: 1.78–12.91, *P* = .025), stage 5 CKD (AOR 4.03, CI: 1.17–13.73, *P* = .016), and CKD duration of less than 1 year (AOR 3, CI: 1.19–9.11, *P* = .007) were significantly associated with anemia. The prevalence of anemia among stage 3 to 5 CKD patients was very high. Anemia was significantly associated with the severity and duration of CKD. Therefore, serial follow-up of patients with a long duration and advanced stages of CKD may help prevent anemia and its adverse consequences.

## 1. Introduction

Chronic kidney disease (CKD), defined as abnormalities in kidney structure or function, present for greater than 3 months,^[1]^ is a complex global public health problem that affects approximately 500 million individuals worldwide, of which more than 3 to 4^th^ reside in developing countries.^[2]^ Progressive CKD is associated with all-cause and cardiovascular mortality, anemia, infections, acute kidney injury, mineral and bone disorders, and hospitalization.^[1,3]^

Anemia is a common complication of CKD and is associated with adverse outcomes, including cognitive impairment, sleep disturbances, CKD progression, cardiovascular, and cerebrovascular events, increased risk of hospitalization, all-cause mortality, and reduced quality of life.^[4,5]^ According to the kidney disease improving global outcomes anemia guidelines, anemia in CKD is considered when the hemoglobin (Hgb) level is <13 g/dL in men and <12 g/dL in women, and CKD is considered a possible cause of anemia when the glomerular filtration rate (GFR) is <60 mL/minutes/1.73m^2^.^[6,7]^ The causes of anemia in patients with CKD are complex and multifactorial. They include a decrease in endogenous erythropoietin (EPO) production, absolute and/or functional iron deficiency, and inflammation with increased levels of hepcidin. Insufficient erythropoietin production results in a decrease in the signaling molecules that stimulate red blood cell production. On the other hand, increased hepcidin levels are known to block iron release from iron stores and its absorption from food in the intestine. Suppression of erythropoiesis due to severe infectious processes, inflammatory diseases, malignancies, and uremic toxins are other possible causes of anemia associated to CKD.^[8,9]^

Studies have shown a wide variation in the prevalence of anemia in CKD across regions. For instance, the reported prevalence of anemia in CKD is 14% in the USA,^[10]^ 39.36% in India,^[11]^ 51.5% in China,^[12]^ 43.18% in South Africa,^[13]^ and 79% in Cameroon.^[14]^ Moreover, the prevalence increases with the CKD stage, with an overall prevalence of 22.4%, 41.3%, and 53.9% in CKD stages 3, 4, and 5, respectively.^[15]^

In Ethiopia, little is known about the prevalence of anemia in patients with CKD. Studies conducted in Addis Ababa and Gondar, northwest Ethiopia showed that the overall prevalence of anemia is 53.5% and 64.5%, respectively. A history of hemodialysis, female sex, and rural residence are factors associated with anemia in patients with CKD.^[16,17]^ The use of iron therapies and erythropoiesis-stimulating agents (ESAs) has been shown to improve outcomes in CKD patients with anemia. However, the costs of ESA treatment, limited availability in resource-limited settings, treatment-related risks such as cardiovascular and cancer risks, and unknown optimal dose complicate treatment.^[6,7,18]^ Therefore, identifying risk factors and preventing modifiable determinants may help avoid the adverse outcomes of anemia in patients with CKD.^[19]^ This study aimed to determine the prevalence of anemia and its predictors among stage 3 to 5 CKD patients admitted to the Jimma medical center (JMC), Southwest Ethiopia.

## 2. Methods

### 2.1. Study design, setting, and population

This institution-based cross-sectional study was conducted at the JMC between September 1 and November 30, 2020. JMC is located in Jimma town, Oromia region, southwest Ethiopia, 346 km from the capital, Addis Ababa. It is the main teaching and referral center in Southwest Ethiopia, providing both inpatient and outpatient services for approximately 15 million people. The hospital has a medical department with an emergency medical outpatient department, general OPD, subspecialty clinics, dialysis unit, and medical ward with 120 beds. Patients with CKD are seen at emergency medical outpatient department and general OPDs, renal clinics, dialysis units, and medical wards. All stage 3 to 5 CKD patients above 18 years of age who were willing to provide written informed consent were included in the study. Patients on dialysis, with a known cause of anemia other than CKD, known malignancy, pregnant women, and those who were not willing to participate were excluded from the study. The sample size was determined by using single population proportion formula with the prevalence of 64.5% as reported from a study done in Gondar,^[15]^ at a 95% confidence interval and 5% margin of error. Consecutive sampling was used to recruit 150 patients in to the study.

### 2.2. Definitions

CKD was defined according to the kidney disease improving global outcomes criteria as abnormalities of kidney structure or function, present for greater than 3 months, with implications for health, and classified based on cause and GFR category (G3–G5).^[1]^ GFR was determined by using CKD-EPI equation for eGFR, stage 3, eGFR 30 to 59 mL/minutes/1.73m^2^; stage 4, eGFR 15 to 29 mL/minutes/1.73m^2^; and stage 5, eGFR <15 mL/minutes/ 1.73m^2^. Anemia was defined as serum hemoglobin levels ≤12 g/dL in women and ≤13 g/dL in men and its severity was classified as severe (<8 g/dL), moderate (8–11g/dL), and mild (11–11.9g/dL) for females and 11 to 12.9g/dL for males.^[6,20]^ Proteinuria was defined as presence of albumin in urine.^[1,17]^

### 2.3. Data collection and procedure

Data were collected by trained health care professionals using an interviewer-administered pretested structured questionnaire to obtain sociodemographic data and a data abstraction checklist to collect relevant information (medical history, laboratory parameters, and drug lists) from patient medical records. Data collectors and patients have applied standard COVID-19 infection preventive measures, such as wearing face masks, hand washing, and maintaining an adequate distance. Data collectors measured the patients’ weight (in kilograms) and height (in meters) to calculate body mass index. A nurse collected approximately 2 to 3 mL of blood sample in 5mL of chemistry test tubes for each patient with anemia to determine serum ferritin levels. The samples were analyzed using a Beckman Coulter AU chemistry analyzer. The complete blood counts (CBC) and peripheral morphology were also determined. The hemoglobin level was used to define anemia and classify the severity of anemia. The serum creatinine was used to estimate GFR using the CKD-EPI equation. All the questionnaires were checked daily for completeness and consistency.

### 2.4. Data processing and analysis

Data were entered into the Epi data manager version 4.4.1 and then exported to SPSS version 22 (IBM Corp., Armonk, NY) for analysis. Data are presented as mean ± standard deviation for continuous variables and as frequency and proportions for categorical variables. The chi- square test was used to compare the proportions of categorical variables. A logistic regression model was used to determine the factors associated with anemia in patients with CKD. Odds ratios (OR), *P* values, and 95% confidence intervals (CIs) were used to determine associations. Variables with a *P* value of less than 0.2 from the bivariable analysis were included in the multivariable analysis. Statistical significance was considered at a *P* value of less than .05 on multivariable logistic regression.

## 3. Results

### 3.1. Socio-demographic characteristics of patients

A total of 150 patients were included in this study. Of these, 97 (64.67%) were male and 90 (60%) were rural dwellers. The mean (±standard deviation) age was 45.34 (±15.17) years, and 70 (46.6%) of them were within the age range of 40-64 years. More than half, 84 (56.0%) of the patients had no formal education, 99 (66%) were Muslims,124 (82.7%) were married, and 59 (39.3%) were self-employed (Table 1).

**Table 1:** Sociodemographic characteristics of stage 3–5 CKD patients, JMC, 2021.

Variable	Category	Frequency (n)	Percent (%)
Sex	Male	97	64.67
	Female	53	35.33
Age in yr	18–39	58	38.7
	40–64	70	46.6
	≥ 65	22	14.7
Residence	Urban	60	40
	Rural	90	60
Marital status	Single	22	14.7
	Married	124	82.6
	Divorced	3	2
	Widowed	1	0.7
Religion	Muslim	99	66
	Orthodox Christian	40	26.7
	Protestant Christian	9	6
	Others	2	1.3
Educational status	Illiterate	84	56
	Elementary school	24	16
	High school	21	14
	College and above	21	14
Occupation	Self-employed	59	39.3
	Farmer	48	32
	Business	26	17.3
	Civil servant	17	11.3
Monthly income (ETB)	<1000	53	35.3
	1000–5000	72	48
	>5000	25	16.7

CKD = chronic kidney disease, ETB = Ethiopian birr, JMC = Jimma medical center.

### 3.2. Clinical characteristics of the patients

Of the total study participants, approximately 1 to 5^th^, 31 (20.7%) had a history of smoking, more than half, 77 (51.68%) had a body mass index label of 18.5 to 24.9, and 3 to 4^th^, 112 (74.7%) had proteinuria. Thirty-six (24%) patients had underlying comorbidities. Heart Failure, 20 (13.3%), benign prostatic hyperplasia, 5 (3.3%), diabetes, 3 (2%), hypertensive heart disease 2 (1.3%), hypertension 2 (1.3%), tuberculosis 2 (1.3%), chronic liver disease 1 (0.7%), and fatty liver disease 1 (0.7%) were common underlying comorbidities. Among the study participants, 85 (56.67%) had stage 5 CKD followed by stage 4,39 (26.0%) and stage 3,26 (17.33%). Most patients, 99 (66%) had a CKD duration of fewer than 6 months. Hypertension, 61 (40.7%), diabetes, 22 (14.7%), obstructive uropathy, 5 (3.3%), and glomerular disease, 3 (2%) were the most common causes of CKD. The remaining 59 (39.3%) had CKD of unknown cause (Table 2).

**Table 2:** Clinical characteristics of CKD patients at JMC, Ethiopia 2021.

Variable	Category	Frequency(n)	Percent (%)
History of smoking	Yes	31	20.7
	No	119	79.3
BMI	<18.5	21	14
	18.5–24.9	77	52
	25–29.9	3	2
	≥30	3	2
	Unknow	45	30
Comorbidity	Present	36	24
	Absent	114	76
Proteinuria	Present	112	74.7
	Absent	38	25.3
Stage of CKD	3	26	17.33
	4	39	26
	5	85	56.67
Cause of CKD	Diabetes	22	14.7
	Hypertension	61	40.7
	Glomerular disease	3	2
	Obstructive Uropathy	5	3.3
	Unknown	59	39.3
Duration of CKD	<6 mo	99	66
	6 mo–1 yr	18	12
	1 yr–5 yr	25	16.7
	>5 yr	8	5.3

BMI = body mass index, CKD = chronic kidney disease, JMC = Jimma medical center.

### 3.3. Prevalence and patterns of anemia among stage 3 to 5 CKD patients

**Figure 1.**
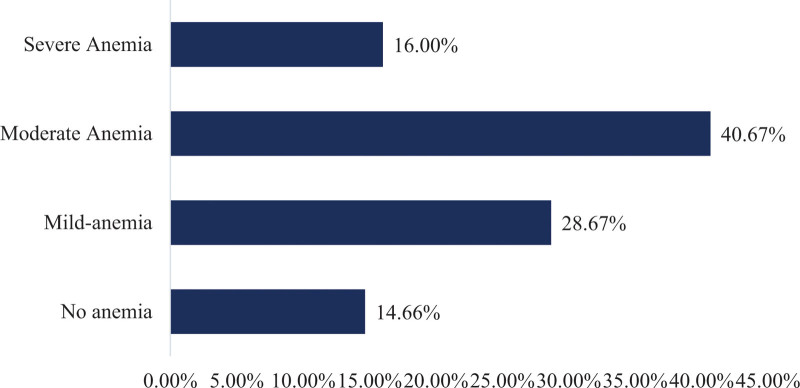
Magnitude and severity of anemia among CKD patients at JMC, Ethiopia, 2021. anemia was defined as hemoglobin level ≤12 g/dL in females and ≤ 13 g/dL in males). Mild anemia (hemoglobin level of 11–11.9 g/dL for females and 11–12.9 g/dL for males), moderate anemia (hemoglobin level of 8–11g/dL), and severe anemia (hemoglobin level < 8 g/dL). CKD = chronic kidney disease, JMC = jimma medical center.

Overall, 1 hundred 28 (85.34%) patients were found to have anemia. Of this, 61 (47.65%) had iron deficiency anemia. Of these patients, 43 (28.67%), 61 (40.67%), and 24 (16%) had mild, moderate, and severe anemia, respectively (Fig. 1). The prevalence of anemia was proportional to CKD stage: - 79 (52.67%), 29 (19.33%), and 20 (13.33%) had stage 5, stage 4, and stage 3 CKD, respectively. The severity of anemia also varied across CKD stages and a higher percentage of severe anemia was found in stage 5 CKD, 17 (11.33%), followed by stage 3, 5 (3.33%), and stage 4, 2 (1.33%) CKD. Furthermore, moderate and severe stages of anemia were more common in patients with hypertension as a cause of CKD, CKD duration of greater than 1 year, and underlying comorbidities (Table 3). Concerning the treatment of anemia, 54 (42.18%) patients received oral iron therapy, and 35 (24.48%) patients received a blood transfusion.

**Table 3:** Clinical characteristics of patients stratified by severity of Anemia.

		Severity of Anemia		
Variables	Category	Mild (n = 43)	Moderate (n = 61)	Severe (n = 24)
Stage of CKD	Stage 3	11 (25.6%)	4 (6.6%)	5 (20.8%)
	Stage 4	16 (37.2%)	11 (18%)	2 (8.3%)
	Stage 5	16 (37.2%)	46 (75.4%)	17 (70.8%)
Cause of CKD	Hypertension	17 (39.5%)	28 (45.9%)	9 (37.5%)
	Diabetes	6 (14%)	7 (11.5%)	3 (12.5%)
	Obstructive Uropathy	2 (4.7%)	3 (4.9%)	-
	Glomerular disease	2 (4.7%)	1 (1.6%)	-
	Unknown cause	16 (37.2%)	22 (36.1%)	12 (50%)
Duration of CKD	<6 mo	21 (48.8%)	24 (39.3%)	9 (37.5%)
	6 mo–1 yr	11 (25.6%)	24 (39.3%)	10 (41.7%)
	1 yr–5 yr	9 (20.9%)	12 (19.7%)	5 (20.8%)
	>5 yr	2 (4.7%)	1 (1.6%)	-
Co-morbidity	Present	15 (34.9%)	10 (16.4%)	5 (20.8%)
	Absent	28 (65.1%)	51 (83.6%)	19 (79.2%)
Proteinuria	Present	30 (69.8%)	48 (78.7%)	17 (70.8%)
	Absent	13 (30.2%)	13 (21.3%)	7 (29.2%)

CKD = chronic kidney disease.

### 3.4. Factors associated with anemia among stage CKD patients

The factors associated with anemia are shown in Table 4. Univariate logistic regression analysis revealed that: - age, sex, residence, CKD stage, CKD duration, and underlying comorbidities were associated with anemia. On multivariate logistic regression analysis, patients with stage 4 CKD (adjusted odds ratio [AOR] 3.2, CI: 1.78–12.91, *P* = .025) and stage 5 CKD (AOR 4.03, CI: 1.17–13.73, *P* = .016) were approximately 3 times and 4 times at higher risk of anemia, respectively, then patients with less severe CKD. Patients with CKD duration greater than or equal to 1 year (AOR 3, CI: 1.19–9.11, *P* = .007) had 3 times higher risk of anemia than patients with a duration of CKD less than 1 year.

**Table 4:** Bivariable and multivariable logistic regression analysis of factors associated with anemia among stage 3–5 CKD patients at JMC, Ethiopia.

		Bivariate logistic regression		Multivariate logistic regression	
Variables	Category	OR (95% CI)	*P* value	AOR (95% CI)	*P* value
Age in yr	18–39	Reference		Reference	
	40–64	10.8 (1.33, 87.6)	.026	0.3 (0.026, 2.411)	.231
	≥65	3.8 (1.46, 32.38)	.012	2.7 (0.848, 8.294)	.094
Sex	Male	Reference		Reference	
	Female	0.4 (0.15, 0.98)	.046	0.23 (0.07, 1.71)	.054
Residence	Urban	Reference		Reference	
	Rural	2.5 (1.002, 6.53)	.05	2.6 (0.86, 7.98)	.089
Duration of CKD	< 1	Reference		Reference	
	≥ 1 yr	2.8 (1.11, 7.25)	.029	3 (1.19, 9.11)	.007*
Stage of CKD	Stage 3	Reference		Reference	
	Stage 4	3.9 (1.15, 13.55	.029	3.2 (1.78, 12.91)	.025*
	Stage 5	4.5 (1.54, 13.62)	.007	4.03 (1.17, 13.73)	.016*
Comorbidity	Yes	2.2 (1.18, 5.49)	.009	2.8 (0.89, 8.63)	.076
	No	Reference		Reference	

CI = confidence interval, CKD = chronic kidney disease, JMC = Jimma medical center.

* Statistically significant.

## 4. Discussion

Anemia is a common complication in patients with CKD and has a wide range of adverse consequences including, reduced renal function and CKD progression, increased cardiac output, left ventricular hypertrophy, angina, congestive heart failure, and reduced survival.^[21]^ Therefore, identifying risk factors and minimizing modifiable determinants may help prevent anemia and its adverse outcomes in CKD patients.^[19]^ In our study, we found that the overall prevalence of anemia among stage 3 to 5 CKD patients was 85.33%. This is higher than the findings reported in previous studies conducted in Ethiopia, 53.5% in Addis Ababa^[16]^ and 64.5% in Gondar, northwest Ethiopia.^[17]^ This result is also higher than the 43.18% reported in a study conducted in South Africa^[13]^ and the 39.36% in India.^[11]^ The variation with these studies might be due to differences in the study population that is, the inclusion of patients with advanced stages of CKD that is, stage 3 and above in our study, the study setting (single vs multi-center), and the number of patients included in these studies. Moreover, the high prevalence of anemia demonstrated in this study may be due to the high number of patients with advanced stage CKD. Our study revealed that iron deficiency anemia was present in nearly half (47.65%) of the patients with anemia. This is higher than the results reported in other studies, 39% in India,^[11]^ 36.3% in Spain, and Catalonia.^[22]^ Overall, the treatment of patients with anemia in this study was suboptimal. Only 42.18% of the patients received oral iron therapy, 24.48% received a blood transfusion, and none of the patients received ESA due to limited availability and unaffordable costs.

A study conducted in Gondar revealed that approximately 55% of all patients with anemia received iron therapy, 55% received blood transfusion, and 14.3% received ESA.^[17]^ In our study the most common causes of CKD were hypertension and diabetes mellitus. This is similar to the results reported in previous studies.^[16,22]^ Hypertension is both a cause and a complication of CKD and diabetes is a common cause of CKD. Therefore, appropriate control of blood pressure and blood glucose level may help to slow the progression of CKD, prevent its complications including anemia, improve survival, and reduce healthcare costs associated with the disease.^[23]^

Many studies have shown an association between CKD stage and the prevalence and severity of anemia.^[10,16,24,25]^ Our study found that the CKD stage was significantly associated with the prevalence of anemia (*P* < .05). More than half (52.67%) of the anemic patients had stage 5 CKD, and the prevalence of anemia increased with CKD severity. This is consistent with several other studies conducted in Ethiopia,^[17]^ Nigeria,^[26]^ the USA,^[10]^ Spain, Catalonia.^[22]^ In a study conducted in patients with non-dialysis CKD, the prevalence of anemia in patients with stage 5 CKD was 51%.^[4]^ It has been shown that a decline in GFR is associated with a decrease in hemoglobin levels and a subsequent increase in the incidence and prevalence of anemia.^[8]^ Accordingly, to identify CKD patients at high risk for anemia, hemoglobin level monitoring should be more frequent in proportion to CKD stages, at least annually in patients with stage 3 CKD and twice a year in patients with CKD stage 4 to 5, not on dialysis.^[1,7]^ Progressive CKD is associated with numerous complications including anemia, which occurs with greater severity in the advanced stages of the disease and cause adverse clinical consequences.^[5]^ Our study found that patients with a long duration of CKD (CKD duration of greater than 1 year) had a 3 times higher risk of anemia (AOR 3, CI: 1.19–9.11, *P* = .007) than patients with a short duration of CKD. Therefore, patients with persistent CKD should be assessed for the presence of anemia and receive optimal treatment to prevent adverse outcomes including CKD progression associated with anemia.^[27]^

Our study has some limitations. First, this was a single-center study and included only patients with advanced stages of CKD (stage 3–5) which might have overestimated the prevalence of anemia. Second, this was a cross-sectional study, thus, a longitudinal study is recommended to assess the factors associated with or resulting from CKD.

In conclusion, the prevalence of anemia among patients with CKD is very high and increases with CKD severity. Advanced stage and long CKD duration were independently associated with anemia. Therefore, identifying patients with advanced stages and long CKD duration may help prevent anemia and its subsequent adverse consequences.

## Acknowledgments

We are grateful to the staff of the hospital, study participants, and data collectors for their cooperation which enabled successful completion of the study.

## Author contributions

**Conceptualization:** Filagot Bishaw, Maekel Belay Woldemariam, Gashahun Mekonen, Bezawit Birhanu, Abinet Abebe.

**Data curation:** Filagot Bishaw, Maekel Belay Woldemariam, Gashahun Mekonen, Bezawit Birhanu, Abinet Abebe.

**Formal analysis:** Filagot Bishaw, Maekel Belay Woldemariam, Gashahun Mekonen, Bezawit Birhanu, Abinet Abebe.

**Funding acquisition:** Filagot Bishaw, Maekel Belay Woldemariam, Gashahun Mekonen, Bezawit Birhanu, Abinet Abebe.

**Investigation:** Filagot Bishaw, Maekel Belay Woldemariam, Gashahun Mekonen, Bezawit Birhanu, Abinet Abebe.

**Methodology:** Filagot Bishaw, Maekel Belay, Gashahun Mekonen, Bezawit Birhanu, Abinet Abebe.

**Project administration:** Filagot Bishaw, Maekel Belay Woldemariam, Gashahun Mekonen, Bezawit Birhanu, Abinet Abebe.

**Resources:** Filagot Bishaw, Maekel Belay Woldemariam, Gashahun Mekonen, Bezawit Birhanu, Abinet Abebe.

**Software:** Filagot Bishaw, Maekel Belay Woldemariam, Gashahun Mekonen, Bezawit Birhanu, Abinet Abebe.

**Supervision:** Filagot Bishaw, Maekel Belay Woldemariam, Gashahun Mekonen, Bezawit Birhanu, Abinet Abebe.

**Validation:** Filagot Bishaw, Maekel Belay Woldemariam, Gashahun Mekonen, Bezawit Birhanu, Abinet Abebe.

**Visualization:** Filagot Bishaw, Maekel Belay Woldemariam, Gashahun Mekonen, Bezawit Birhanu, Abinet Abebe.

**Writing – original draft:** Filagot Bishaw, Maekel Belay Woldemariam, Gashahun Mekonen, Bezawit Birhanu, Abinet Abebe.

**Writing – review & editing:** Filagot Bishaw, Maekel Belay Woldemariam, Gashahun Mekonen, Bezawit Birhanu, Abinet Abebe.
